# Identifying Sex Differences in Adverse Events Reported on Opioid Drugs in the FDA’s Adverse Event Reporting System (FAERS)

**DOI:** 10.3390/ph19040526

**Published:** 2026-03-25

**Authors:** Aasma Aslam, Huixiao Hong, Tucker A. Patterson, Wenjing Guo

**Affiliations:** National Center for Toxicological Research, US Food and Drug Administration, Jefferson, AR 72079, USA; aasmaaslam@yahoo.com (A.A.); huixiao.hong@fda.hhs.gov (H.H.); tucker.patterson@fda.hhs.gov (T.A.P.)

**Keywords:** drug safety, FAERS, adverse events, sex differences, disproportionate reporting

## Abstract

**Purpose**: Opioids are widely used for pain management but are associated with adverse events that may differ between women and men. However, post-marketing safety data are rarely examined at the individual level to characterize these sex differences. This study aimed to identify sex disparities in opioid-associated adverse events using the FDA Adverse Event Reporting System (FAERS) to inform safer opioid selection for women. **Methods**: Opioid drugs were identified using the FDA’s Opioid Analgesic Risk Evaluation and Mitigation Strategy (REMS) list and official drug labeling. Relevant FAERS reports were extracted, and adverse events were classified into 27 System Organ Classes (SOCs) based on the Medical Dictionary for Regulatory Activities (MedDRA). Sex-specific signals of disproportionate reporting were evaluated using proportional reporting ratios and reporting odds ratios for drug–SOC pairs. **Results**: Across most opioid drugs and SOCs, adverse events were reported more frequently in women than in men. The largest sex disparities were observed for codeine, fentanyl, tapentadol, hydrocodone, and sufentanil, with significantly higher disproportionate reporting rates among women. These findings indicate pronounced sex-specific differences in the post-marketing safety profiles of several commonly used opioids. **Conclusions**: Women demonstrate higher disproportionate reporting of adverse events for certain opioid medications, particularly codeine and fentanyl. These results suggest the need for increased awareness of sex-specific safety differences and support sex-informed prescribing and monitoring strategies to improve opioid safety in women. Since pharmacists are medication experts and play a key role in promoting rational and safe use, our findings may further support pharmacists counseling patients and monitoring for opioid-related adverse events.

## 1. Introduction

Opioids are powerful pain-relieving drugs that include both illegal substances like heroin and legally prescribed drugs such as fentanyl, oxycodone, hydrocodone, and morphine. These drugs are commonly used to manage moderate to severe nociceptive pain in both acute and chronic settings. However, opioid use is associated with significant risks, including misuse, addiction, and a range of adverse events. Although deaths caused by opioid overdose decreased from 83,140 in 2023 to 54,743 in 2024 [1], the mortality rate remains at a high level, reflecting the ongoing opioid crisis, which continues to affect millions worldwide, particularly in the United States.

Emerging evidence suggests that opioid risks may vary across sexes, with women potentially facing a greater vulnerability [2]. Between 1999 and 2020, opioid overdose deaths among women increased nearly tenfold [1]. Several factors may explain this impact. Research has shown that women are more likely to report chronic, severe, and frequent pain compared to men, leading to higher prescription rates, increased doses, and longer treatment durations [3]. This prolonged exposure raises women’s risk of opioid-related adverse events, especially given their heightened sensitivity to certain side effects such as respiratory depression, gastrointestinal issues, and opioid-induced hyperalgesia.

Sex differences in opioid pharmacodynamics and pharmacokinetics suggest that opioids may affect men and women differently due to biological, hormonal, and metabolic factors. Estrogen has been shown to influence opioid receptor activity and pain processing, which may affect analgesic response, dose requirements, and tolerability in women [4,5,6]. During menopausal transition, estrogen levels fluctuate and then decline, and this change is associated with shifts in pain perception and symptom burden. As a result, opioid response may vary across pre, peri, and postmenopausal stages, with potential differences in both analgesic effectiveness and adverse effects [7]. In addition, hormonal fluctuations may influence drug metabolism and clearance, which can alter opioid sensitivity and increase the likelihood of side effects such as sedation, dizziness, and respiratory depression [8].

Respiratory depression is one of the most severe and potentially fatal adverse effects of opioid use and a leading cause of opioid overdose deaths [9]. The greater sensitivity of women to this adverse event is especially concerning for those with underlying conditions like sleep apnea or for individuals concurrently using other central nervous system (CNS) depressants that further slow breathing [10]. In addition, gastrointestinal adverse events such as opioid-induced constipation, nausea, and vomiting are common and can diminish quality of life. Women report gastrointestinal issues more frequently than men, often complicating opioid therapy [11]. CNS-related adverse events, such as sedation and dizziness, are also more prevalent in women, increasing the risk of falls, particularly among older women [12].

Despite these known risks, research on sex-based differences in opioid safety remains limited. Most available studies rely on small clinical trials or narrow patient groups, which may not accurately reflect broader opioid use patterns. To bridge this gap, our study leverages the FDA Adverse Event Reporting System (FAERS) to investigate sex disparities in opioid safety signals. FAERS, a self-reporting database, is widely used to study potential associations between drugs and adverse events [13]. We first collected opioid drug names from the FDA’s Opioid Analgesic Approved Risk Evaluation and Mitigation Strategies (REMS) program database and FDALabel, a database of official drug-labeling documents developed at FDA [14]. We then extracted adverse event data separately for men and women from FAERS using the collected drug names. The extracted adverse events were classified into 27 System Organ Classes (SOCs) according to the Medical Dictionary for Regulatory Activities (MedDRA) for statistical analysis. To quantify drug–adverse event associations, we calculated proportional reporting ratios (PRR) and reporting odds ratios (ROR) for each drug–SOC pair. The calculated PRR and ROR were used to compare the most frequently reported drugs and adverse events between men and women, revealing sex disparities in adverse opioid events. Our findings may improve the safe use of opioids by informing sex disparities in opioid safety profiles, ultimately promoting women’s health.

## 2. Results and Discussion

### 2.1. Opioid Drug Names

Labels of nine pharmacologic classes of drugs were obtained from searching opioids in FDALabel, including 21 labeling documents for competitive opioid antagonists (MOA), 1095 for full opioid agonist MOA (Mechanism of Action), 13 for opioid agonist–antagonist EPC (Established Pharmacologic Class), 1723 for opioid agonists (EPC), 628 for opioid agonists (MOA), 193 for opioid antagonists (MOA), 170 for opioid antagonists (EPC), 107 for partial opioid agonists (MOA), and 86 for partial opioid agonists (EPC). An FDA “Established Pharmacologic Class” (EPC) refers to the pharmacologic class with an approved active moiety that the FDA considers scientifically sound and clinically relevant [15].

From FDALabel entries, we extracted trade names, generic/proper names, and active ingredients. Records with the same values across all three fields were considered duplicates and removed. This process resulted in 349 drug labels, with 80 unique active ingredients. Of the 80 active ingredients, 13 were in salt forms and removed. After the non-opioid active ingredients were discarded, 30 active opioid ingredients were obtained and used in subsequent adverse event identification.

From REMS, we extracted 354 records where the “Product Name” column was used to identify drug names, resulting in 57 names. No additional active opioid ingredients were found in REMS. Of the 57 names, three were not included in the names and synonyms from FDALabel. The final list of 30 opioid drug names and synonyms is provided in Supplementary Table S1.

### 2.2. Adverse Events Extracted

**Figure 1:**
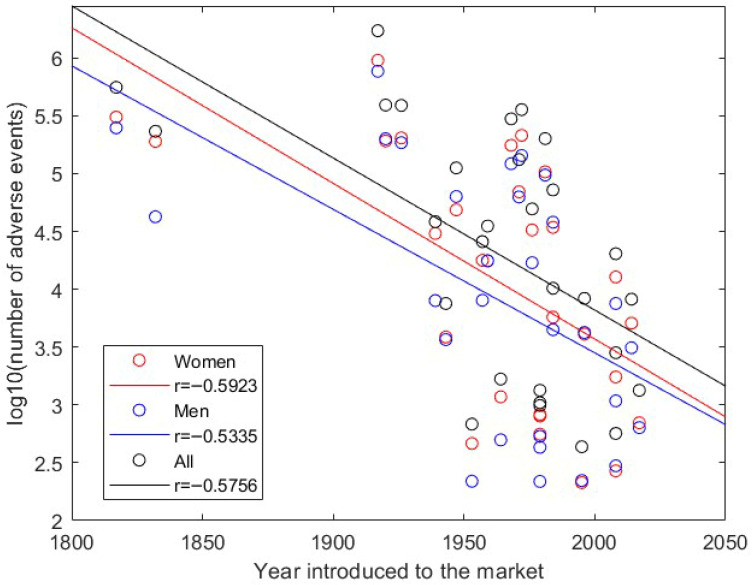
Relationship between number of adverse events and year introduced to the market for opioid drugs. The *x*-axis gives the year introduced to the market and the *y*-axis presents the 10-base logarithmic transformed value of adverse events. Adverse events for women, men, and both are plotted as red, blue, and black circles, respectively, and the corresponding regression results are shown by the red, blue, and black lines.

A total of 5,709,646 adverse events were identified in FAERS (reports from quarter 1 of 2004 to quarter 2 of 2023) using the names and synonyms collected for the 30 opioid drugs (Supplementary Table S2). These adverse events are associated with 29 opioid drugs as no adverse events were found for the opioid drug benzhydrocodone. Benzhydrocodone is a prodrug of hydrocodone, and it was developed to minimize the potential for opioid misuse through non-oral routes like injection or snorting [16]. It is a component of Apadaz, a drug product with the formulation combining benzhydrocodone and acetaminophen, and it was approved by the FDA in February 2018. As shown in Table 1, benzhydrocodone is the most recently introduced drug to the market, and its shorter period of market exposure may limit the accumulation of reported adverse events. Although relevant generic and brand names were searched, the absence of identified reports may still reflect reporting granularity, naming variability, or coding practices in FAERS rather than a true absence of adverse events. Examining the relationship between the dates introducing drugs to the market and adverse events reported in FAERS, shown in Figure 1, revealed moderate negative correlations, with correlation coefficients of −0.5923, −0.5335, and −0.5756 for women, men, and both, respectively, indicating that opioid drugs introduced to the market earlier likely have more adverse events reported in FAERS. Therefore, more adverse events may be reported to FAERS in the future.

Of the extracted adverse events, 18% (1,026,717) were extracted from reports for unspecified sex or transgender individuals and cannot be used to assess sex disparity and thus were not included in subsequent analysis. The remaining 46% (2,631,781) and 36% (2,051,148) were reported for women and men, respectively. The distributions of these adverse events in the opioid drugs and SOCs are summarized in Table 1 and Table 2, respectively. Among the 30 opioid drugs, oxycodone, morphine, and hydrocodone ranked as the top three with 37%, 12%, and 8% of all adverse events, respectively. These three drugs have been on the market for more than 100 years and are the most used opioids in the clinic. “Psychiatric disorders”, “General disorders and administration site conditions”, and “Injury, poisoning and procedural complications” stand out from the 27 SOCs with 27%, 20%, and 10% of the identified adverse events, respectively. Opioid drugs affect brain chemistry and contribute to a physiological state that promotes the development of psychological distress [17], which may contribute to the largest number of adverse events related to psychiatric disorders.

### 2.3. Sex Disparity

**Figure 2:**
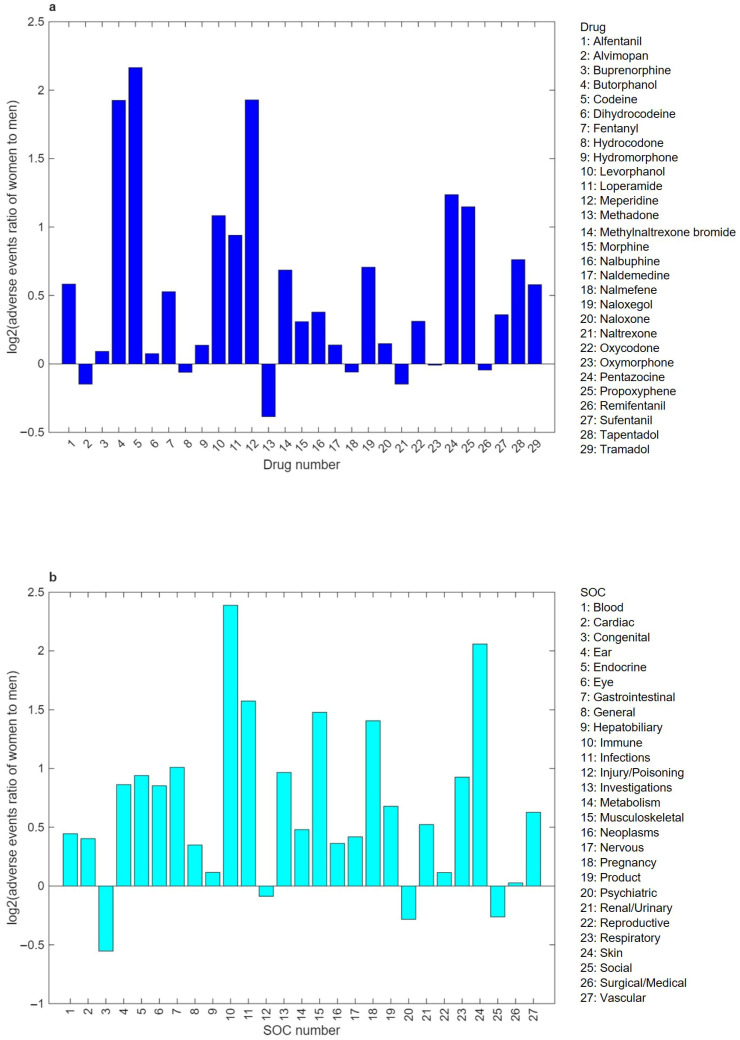
Bar charts for ratios of adverse events reported for women to men across opioid drugs (**a**) and system organ classes (SOCs) (**b**). The log_2_ values of ratios were plotted as bars for opioid drugs and SOCs. The y- and x-axes correspond to the opioid drug numbers and SOC numbers, respectively, as shown in the legends. AEs represent “adverse events”.

To examine sex disparities in adverse events, the ratio of the amounts of extracted adverse events for women to men was calculated for each of the 29 opioid drugs and each of the 27 SOCs. Figure 2 shows log_2_ transformed values of the resulting ratios. As depicted in Figure 2a, more adverse events have been reported for women than men for 23 of the 29 opioids, demonstrating that most opioid drugs have significant sex disparities in adverse events reported in FAERS. For the six opioid drugs that showed more adverse events for men compared to women, the differences are much smaller than those of the drugs having more adverse events for women. It is noteworthy that the sex disparities in adverse events for six drugs (butorphanol, codeine, levorphanol, meperidine, pentazocine, propoxyphene) are considerably large with ratios of >2, indicating that adverse events reported for women are >200% more than for men. Upon closer examination of the number of adverse events, it was revealed that the number of adverse events reported for butorphanol, levorphanol, and pentazocine is very small, though these reports are disproportionately skewed toward women, suggesting that the safety concerns on these three drugs may not be a widespread issue and further research is needed to ensure their comprehensive safety profiles across diverse populations. In contrast, the number of adverse events reported for the other three drugs (codeine, meperidine, propoxyphene) are not only dramatically large but also very skewed to women. The high incidence of adverse events and significant sex disparities indicate major safety concerns of these three drugs, especially for women.

To investigate the variation in sex disparities across different types of adverse events, the ratios comparing the number of adverse events for women to those for men were calculated and are presented in Figure 2b. Similar disparities for opioid drugs were observed for adverse event SOCs. Of the 27 SOCs, adverse events of 23 SOCs have higher reporting rates in FAERS for women than men. It is important to note that adverse events related to “gastrointestinal disorders”, “immune system disorders”, “infections and infestations”, “musculoskeletal and connective tissue disorders”, “pregnancy, puerperium and perinatal conditions”, and “skin and subcutaneous tissue disorders” not only are frequently reported in FAERS, with cases ranging from 44,661 to 297,153, but also are biased to women. Similar sex differences have been reported in clinical and real-world data, where women were more likely than men to report adverse opioid reactions, including gastrointestinal and skin-related events [18]. Sex disparity for the other four types of adverse events (“congenital, familial and genetic disorders”, “injury, poisoning and procedural complications”, “psychiatric disorders”, “social circumstances”) are not substantial, though they are biased to men. Together, these results indicate that sex-related reporting patterns vary by adverse-event domain rather than follow a uniform pattern across all SOCs.

**Figure 3:**
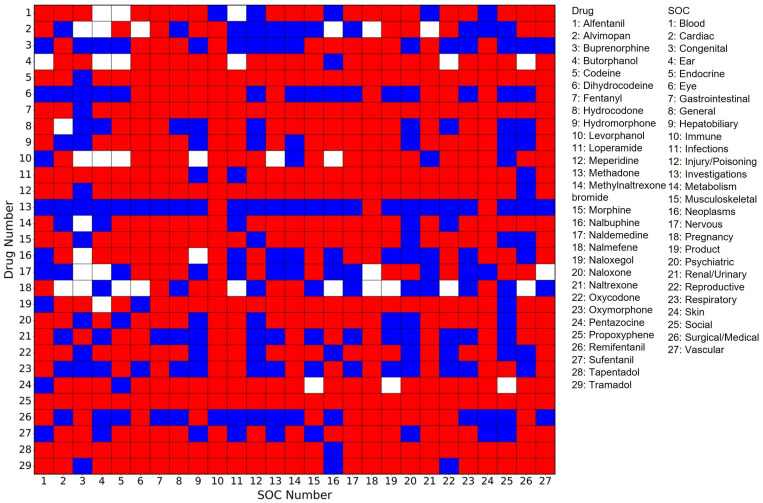
Heatmap of the log_2_ values of ratios of adverse events reported for women to men for the 783 drug–SOC pairs. The y- and x-axes correspond to the opioid drug numbers and SOC numbers, respectively, as shown in the legends. Color codes: red for >0, blue for <0, and white for 0.

Adverse events extracted from FAERS for a given drug are often not evenly distributed across the 27 SOCs. Likewise, adverse events within a specific SOC may not be extracted equally from all 29 opioids. To better understand sex disparities in adverse events in individual SOCs for specific opioid drugs, we calculated the ratios of adverse events reported for women to those for men across all pairs between the 29 opioids and 27 SOCs. The ratios were then transformed into the log_2_ values (Supplementary Table S3) for easy visualization and analysis. To visualize sex disparities and identify drug–SOC pairs having significant disparity concerns, a heatmap of the log_2_ values was generated and is shown in Figure 3—color coded in red for >0, white for =0, and blue for <0. Of the 783 drug–SOC pairs, 522 (64.44%) are in red, indicating that more adverse events have been reported for women than men for most pairs. A closer examination of the heatmap revealed that adverse events in all 27 SOCs associated with propoxyphene are biased toward women, and adverse events in 26 of the 27 SOCs reported for three opioid drugs (codeine, fentanyl, and tapentadol) showed preference for women, indicating that women are more vulnerable to these four opioid drugs. On the other side, adverse methadone events in 24 of the 27 SOCs have been less frequently reported for women than men. Various factors such as faster drug conversion, altered drug distribution, and greater sensitivity to opioid effects may contribute to these sex disparities. For example, codeine is metabolized into morphine, and women’s higher CYP2D6 activity may lead to a faster conversion, increasing potential risks in women [19,20]. Fentanyl’s lipophilicity could cause it to accumulate in women’s higher body fat, leading to prolonged effects and an increased risk of respiratory depression and other severe side effects [21].

Looking more closely at the disparities across the 27 SOCs shown, Figure 3 revealed that adverse events related to immune system disorders (SOC 10) are biased to women for 27 of the 29 opioid drugs, and those related to pregnancy, puerperium, and perinatal conditions (SOC 18) as well as gastrointestinal disorders (SOC 7) are higher for women than men in 26 of the 29 drugs. These results suggest that women are more likely to be affected in their immune system, gastrointestinal tract, and perinatal health when using opioids. Recent research has described the immunomodulatory effects of estrogen and a higher prevalence of certain autoimmune and hypersensitivity conditions in women [22]. While our study does not directly evaluate underlying biological mechanisms, such factors may provide context for the female-biased reporting in immune system-related adverse events. Similarly, pregnancy-related adverse events are inherently female-specific, and previous studies have documented associations between opioid exposure during pregnancy and adverse perinatal outcomes [23,24]. In this context, the observed reporting patterns highlight the importance of considering reproductive status and immune-related conditions when evaluating opioid safety in women.

Several biological mechanisms may contribute to these observed patterns. For example, estrogen has recognized immunomodulatory effects and may influence susceptibility to certain autoimmune and hypersensitivity reactions [25]. Pregnancy-related adverse events are specific to women, and opioid exposure during pregnancy has been associated with neonatal opioid withdrawal syndrome, respiratory depression, and other adverse perinatal outcomes [26,27]. However, because FAERS does not include exposure denominators or detailed pregnancy information, these findings should be interpreted as signals rather than evidence of increased clinical risk.

Lactation is another important consideration. Infant exposure to opioids through breast milk varies across drugs and depends on maternal metabolism. Regulatory agencies recommend avoiding codeine and tramadol during breastfeeding because of the risk of serious respiratory depression in infants, especially in mothers who are CYP2D6 ultra-rapid metabolizers [28]. The disproportionate reporting observed in women, especially for codeine, is, therefore, consistent with existing lactation concerns. Beyond pregnancy and lactation, chronic opioid exposure may affect endocrine function with potential implications for fertility and reproductive health. Opioids can suppress the hypothalamic–pituitary–gonadal axis by reducing gonadotropin-releasing hormone (GnRH) secretion and downstream luteinizing hormone (LH) and follicle-stimulating hormone (FSH) signaling [29,30]. This suppression may lead to menstrual irregularities, reduced libido, and possible fertility effects [29,30,31]. Although FAERS does not capture hormonal measurements or fertility outcomes, these established endocrine mechanisms provide a biological context for interpreting sex-related differences in opioid safety reporting.

### 2.4. Statistical Evaluation of Sex Disparities

The sex disparities for the 783 drug–SOC pairs are in absolute values. Their relative significances are vital for understanding the associations between opioid drugs and adverse events. Therefore, PRR and ROR values for the 783 drug–SOC pairs were calculated based on their sex disparities to statistically evaluate their significances.

**Figure 4:**
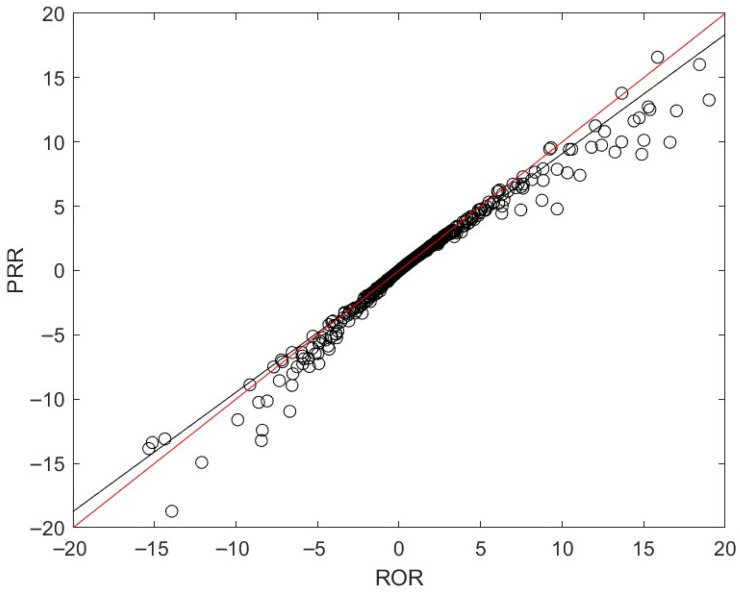
Consistency between ROR and PRR values. The *x*-axis depicts ROR value, and the *y*-axis presents PRR value. The black circles are drug–SOC pairs. The red diagonal line is where ROR and PRR values are the same. The black line is the regression line between the ROR and PRR values using least squares.

Various statistical methods are available, with each having specific advantages and limitations. The characteristics of the dataset under analysis may require a specific statistical method. To assess the consistency between PRR and ROR, their values were compared in Figure 4. For most pairs, their PRR and ROR values are close to the red line, where PRR and ROR should be the same. The black line is the regression line of these drug–SOC pairs by least squares, and it is very close to the red line, also suggesting that most drug–SOC pairs did not exhibit substantial differences in PRR and ROR values. Our results indicate that though different statistical methods vary in metric values, the choice of these methods may not lead to large differences in assessing the significance of sex disparities, as these values correlate and are close to each other.

**Figure 5:**
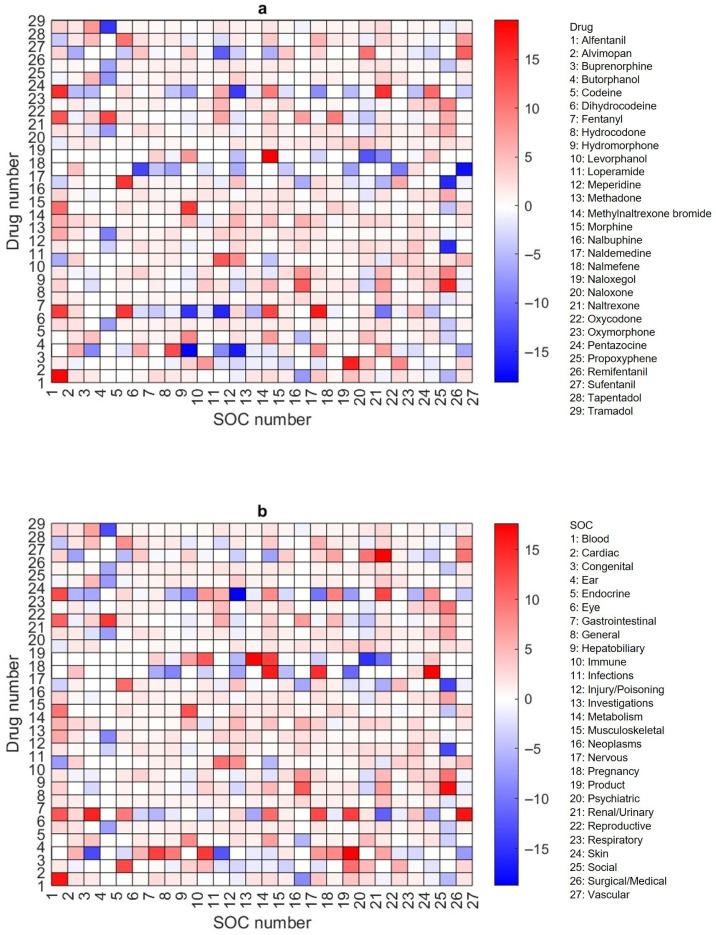
Heatmaps of ROR (**a**) and PRR (**b**) values for the drug–SOC pair by sex: the y- and x-axes correspond to the opioid drug numbers and SOC numbers, respectively, as shown in the legends. The value scale is color-coded, as illustrated by the color bars.

The PRR and ROR values of the 781 drug–SOC pairs are shown in Figure 5. Comparing Figure 5a,b confirmed that PRR and ROR values are very close to each other. A close examination of the PRR and ROR values revealed that many more pairs had significant sex disparity for women than men. More specifically, the sex disparities of 522 (66.7%) and 533 (68.1%) pairs are biased to women (red cells in Figure 5), measured by ROR and PRR, respectively, while only 218 (27.8%) and 207 (26.4%) are biased to men (blue cells in Figure 5), based on ROR and PRR, respectively. Among these pairs, 334 (42.7%) and 345 (44.1%) are significantly biased to women with values greater than or equal to 1 and 154 (19.7%), and 147 (18.7%) are significantly biased to men with values less than or equal to −1, using ROR and PRR, respectively. Examining the distribution of the pairs significantly biased to women on drugs using PRR found that codeine (Drug 5) has the most, with 17 SOCs, followed by hydrocodone (Drug 8) and sufentanil (Drug 27) with 15 SOCs, suggesting that cautions should be taken when prescribing these opioid drugs to women. Interestingly, compared to drugs having the most SOCs of adverse events biased to women (codeine, fentanyl, and tapentadol) showed that codeine not only caused more types of adverse events to women but also showed statistical significance for such sex disparities. The same conclusion was obtained by examining ROR values: codeine, hydrocodone, and sufentanil have the most SOCs of adverse events with significant sex disparities.

Overall, the predominance of adverse event reports in women observed in this study is consistent with prior pharmacovigilance analyses demonstrating that women account for a larger proportion of reported adverse drug reactions across therapeutic classes, including opioids [32,33]. For example, large FAERS-based evaluations have shown higher reporting rates among women even after adjusting for baseline drug utilization [32]. However, most opioid-focused studies have evaluated opioids collectively as a single class or have concentrated on selected adverse events such as overdose mortality, respiratory depression, or neonatal outcomes [34]. In contrast, our analysis assessed sex-related reporting patterns across individual opioid agents and across all 27 SOC categories simultaneously, providing a broader system-level perspective.

At the drug level, prior literature often presents opioids as a homogeneous group when discussing sex differences in safety profiles [35,36]. Our findings demonstrate that reporting imbalance varies among individual opioids, with some drugs showing pronounced female-biased patterns while others exhibit relatively balanced distributions. This heterogeneity is consistent with pharmacologic evidence that opioids differ in metabolism, receptor activity, and adverse effect profiles [37,38]. For instance, variability in the CYP2D6-dependent metabolism of codeine remains a well-established contributor to differences in opioid exposure and toxicity [39]. By examining agents individually, our study extends previous class-level analyses and suggests that sex-related reporting differences may not apply uniformly across all opioids.

At the SOC level, the predominance of psychiatric disorders, general disorders, administration site conditions, and injury/poisoning among reported events aligns with the established clinical understanding of opioid-related adverse effects, including dependence, sedation, and overdose-related complications [40,41,42]. Moreover, the female-biased reporting observed in gastrointestinal, immune system, and pregnancy/puerperium SOCs is consistent with targeted clinical research describing sex differences in opioid tolerability, immune modulation, and perinatal outcomes [38,43,44]. While these prior studies typically focused on individual outcomes, our SOC-level evaluation demonstrates that such sex-related differences appear across multiple physiological domains within a large national reporting system.

The PRR and ROR analyses further showed strong concordance across drug–SOC pairs, supporting the internal consistency of the observed reporting patterns. Although these disproportionality measures are commonly used for drug–event signal detection, in this context, they were applied to quantify relative sex-based reporting imbalances rather than to establish regulatory-level safety signals. The agreement between PRR and ROR reinforces the robustness of the descriptive disparities identified in this dataset.

Taken together, these comparisons indicate that our findings are broadly consistent with the existing evidence of sex-related variability in adverse opioid events while extending prior work by providing drug-level and system-level granularity across the opioid class. Rather than drawing causal conclusions, these results add real-world context that may inform future exposure-adjusted and clinically detailed investigations of sex-specific opioid safety. Future studies leveraging prescription databases, insurance claims data, or electronic health records will be important to validate these reporting patterns and better quantify sex-specific differences in opioid safety.

Several limitations should also be considered when interpreting our findings. Since this analysis was conducted at the SOC level and did not differentiate specific adverse event subtypes or stratify by seriousness, it limits the detailed clinical interpretation of individual adverse event profiles. Furthermore, this study is based on spontaneous reports from FAERS, the results show reporting patterns rather than confirmed incidence rates, and causal relationships cannot be established using the data. As a passive surveillance system, FAERS is subject to underreporting and reporting bias. The reporting frequency of an adverse event may vary depending on drug characteristics, event severity, time period, and the awareness of patients or healthcare professionals. FAERS also lacks detailed clinical information. Important variables such as dosage, treatment duration, comorbidities, concomitant medications, pregnancy status, and hormonal stage are often missing or inconsistently reported. The absence of these data limits the ability to adjust for confounding factors and to fully interpret observed sex-related differences. Combination products were analyzed at the ingredient level to support consistent signal detection, but our approach does not determine which specific component contributed to a reported event.

Despite these limitations, the large number of reports and the consistent sex-related patterns observed across multiple opioid drugs support the relevance of our findings. These results suggest that sex differences should be considered in post-marketing opioid safety evaluation. They also provide hypothesis-generating evidence and support further studies using more detailed clinical and exposure data to better understand sex-specific opioid safety.

## 3. Materials and Methods

### 3.1. Study Design

**Figure 6:**
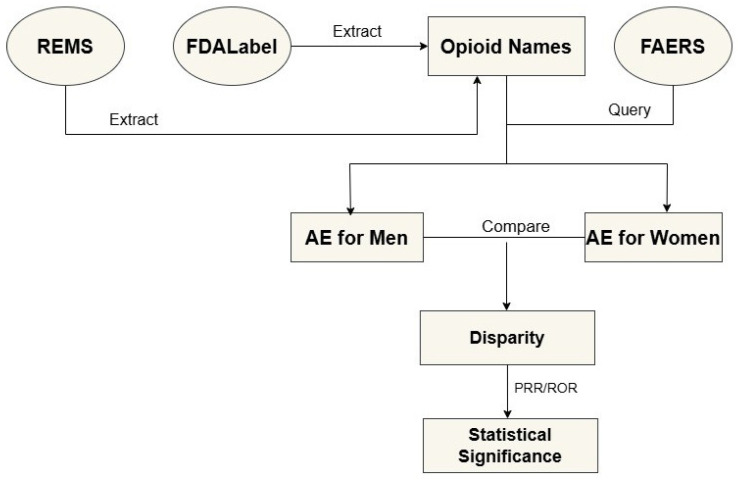
Study design. The flowchart illustrates this study, including the collection of opioid drug names and synonyms, extraction of adverse events from FAERS, calculation of sex disparities, and assessment of statistical significance of the sex disparities.

The study workflow is presented in Figure 6. First, opioid names and their synonyms were collected from the FDA’s Opioid Analgesic REMS program [45] and FDALabel databases [14]. These opioid names and synonyms were then used to search the FDA’s FAERS database for associated adverse events. The identified adverse events were categorized into 27 SOCs and separated by sex. Sex disparities in adverse events were calculated for the drugs and SOCs as well as drug–SOC pairs. The statistical significance for the sex disparity of each drug–SOC pair was evaluated using PRR and ROR.

### 3.2. Opioid Drug Names

Opioid names and their synonyms were collected from FDALabel and the FDA’s Opioid Analgesic REMS program database. The FDALabel database is a publicly available resource containing information on over 150,000 official drug-labeling documents, covering human prescription drugs, over-the-counter medications, and biologics. The FDA’s Opioid Analgesic REMS program educates healthcare providers on managing opioid treatments to mitigate safety risks. Using both sources, a comprehensive list of opioid drug names and their synonyms was compiled.

Within FDALabel, two labeling types (human prescription drugs and human over-the-counter medications) were searched and the keyword “opioid” for pharmacologic classes was used to identify drug labels containing opioids. Since the search strategy was based on pharmacologic class, opioid receptor–active agents beyond centrally acting analgesics were included. For example, loperamide, a peripherally acting μ-opioid receptor agonist primarily used as an antidiarrheal agent, was included in the analysis. Drug names were extracted from the columns “trade name”, “generic/proper name(s)”, and “active moiety name(s)” in the resulting documents downloaded separately for each pharmacologic class. Labels with identical values across all three columns were considered duplicates and removed. We removed salt forms from active ingredient names, including dihydrate, bitartrate, methylbromide, hydrochloride, hydrobromide, chloride, bromide, citrate, sulfate, sulphate, oxalate, tosylate, acetate, maleate, hydrate, mesylate, tartrate, napsylate, phosphate, and succinate. For drugs containing multiple active ingredients, we separated each component and assigned trade, generic, and proper names to each individual ingredient. For example, “oxycodone/naloxone” was split into “oxycodone” and “naloxone”, and the trade name Targin was assigned to both. The non-opioid components (e.g., acetaminophen, acetylsalicylic acid) were excluded from the active opioid ingredient list.

Drug names in the “Product Name” field of the REMS database were extracted. The same processing approach was applied to the resulting list of drug names. The opioid drug names extracted from REMS and FDALabel were then combined. If the same name was extracted from both databases, only one was kept. This process resulted in a final list of 30 opioids with generic names and their synonyms as given in Table 1, which we used to search the FAERS database for adverse event reports.

### 3.3. Adverse Event Extraction

FAERS is a self-reporting system that collects adverse events associated with drugs. It is widely used in pharmacovigilance studies. Quarterly FAERS data files were downloaded covering the period from the first quarter of 2004 to the second quarter of 2023. Data were extracted, including patient sex from the “sex” column in the demo.txt file, drug names from the “drugname” column in the drug.txt file, and adverse events from the “reac.txt” file. These files were linked using the primary report ID as a unique identifier.

Reports with missing, blank, or unspecified sex entries were excluded from the analysis to ensure accurate sex-based comparisons. Some entries were duplicates or incomplete. Thus, to improve data quality, duplicate and incomplete records were removed. For duplicate reports with the same case ID, drug name, and adverse event, only the latest record was retained. Since narrative text and additional patient identifiers are not available in the publicly released FAERS data, advanced probabilistic de-duplication methods cannot be reliably implemented. Probabilistic matching using only limited structured variables (e.g., age, sex, country) may introduce misclassification. Therefore, rule-based de-duplication was applied, and residual duplicate reports cannot be completely excluded. Records missing critical details, such as case ID, drug name, or adverse event, were excluded. To reduce false positives, we only included reports that labeled the drugs as “primary suspected” and “secondary suspected”.

FAERS reports use opioid names in various forms, including trade names, generic names, and abbreviations. Both generic names and synonyms were used to ensure comprehensive extraction of relevant adverse events. Adverse events in FAERS are recorded using MedDRA’s preferred terms or lowest level terms. In this study, MedDRA version 22.1 was used to classify adverse events into 27 SOCs (Table 2). When a term was mapped to multiple SOCs, only primary SOCs were considered, and unmatched terms were excluded. MedDRA version updates may introduce minor classification changes; these are unlikely to substantially affect SOC-level analyses based on large aggregate datasets.

### 3.4. Sex Disparity in Adverse Events

To assess sex disparity in adverse events associated with opioids, the identified adverse events were separated by the “sex” field in the FAERS demographic data. The numbers of adverse events reported for women and men were compared for each drug–SOC pair. Specifically, the ratio of adverse events for women to those for men was calculated and this ratio was then log_2_ transformed. A log_2_ > 0 means women have more adverse events reported than men, a log_2_ < 0 indicates men have more adverse events reported than women, and a zero log_2_ implies adverse events are equally reported for both sexes. When no adverse events were identified for women, the ratio was zero and a log_2_ of −5 was assigned as the lowest log_2_ for non-zero ratios was −4.6. When no adverse events were identified for men, the ratio was infinite and a log_2_ of 6 was assigned because the largest log_2_ is 5.1. If no adverse events were identified for both men and women, a log_2_ zero was set to indicate no sex disparity.

### 3.5. PRR and ROR Calculations

To evaluate statistical significance of the sex disparities in adverse events, PRR [46] using Equation (1) and ROR [47] using Equation (2) were calculated for the drug–SOC pairs. PRR is a statistical measurement that compares the ratio of the sex disparity of a particular drug–SOC pair to the disparities of pairs between the same drugs and other SOCs with the ratio of the sex disparities pairs between the same SOC and other drugs to the sex disparities of pairs between other drugs and other SOCs. ROR is a disproportionality measure that compares two ratios: one is the ratio of the sex disparity of a drug–SOC pair to the sex disparities of pairs between the drug and other SOCs, and another is the ratio of the sex disparities of pairs between the SOC and other opioid drugs to the sex disparities of pairs between other opioid drugs and other SOCs. In this study, PRR and ROR were applied to assess sex-based reporting differences rather than traditional drug–event signal detection. Therefore, these measures should be interpreted as indicators of relative reporting imbalance rather than regulatory signal detection metrics. Statistical thresholds were used to improve robustness; however, results are intended for comparative pattern analysis rather than formal signal identification. For a drug–SOC pair, a PRR > 1 or ROR > 1 suggests that the sex disparity of the drug–SOC pair is more biased to women compared to the pairs between the same SOC and other drugs. A PRR < 1 or ROR < 1 indicates that the sex disparity of the drug–SOC pair is less biased to women compared to the pairs between the same SOC and other drugs. A PRR = 1 or ROR = 1 indicates that the sex disparity of the drug–SOC pair is at the same level compared to the pairs between the same SOC and other drugs.

(1) $$PRR=(\frac{a}{a+b})/(\frac{c}{c+d})\;$$

(2) $$ROR=\frac{\left(a\;*\;d\right)}{\left(b\;*\;c\right)}$$

where *a* is the sex disparity for a specific drug–SOC, *b* is the sex disparities of pairs between the same drug and other SOCs, *c* the sex disparities of pairs between the same SOC and other drugs, and *d* is the sex disparity of pairs between other drugs and other SOCs.

### 3.6. Sex Disparities Assessment

The opioid–SOC pairs were categorized into three groups based on their log_2_ PRR or log_2_ ROR values using thresholds commonly employed in pharmacovigilance studies to indicate the difference in statistical significance [48]. A log_2_ PRR or log_2_ ROR value greater than 1 corresponds to PRR or ROR > 2, while a value less than −1 corresponds to PRR or ROR < 0.5. The pairs with log_2_ PRR or log_2_ ROR values greater than 1 indicate that adverse events of the SOCs have been reported for the corresponding opioids more frequently than for other opioids in FAERS. On the contrary, values smaller than −1 denote that adverse events of the SOCs are less for the corresponding opioids than for other opioids. Values between −1 and 1 imply that adverse events of the SOCs for the corresponding opioids are not statistically different from those for other opioids.

All data processing and statistical analyses were performed using in-house scripts in Python 3.6 (Python Software Foundation, http://python.org (accessed on 1 June 2025)) (Python Software Foundation, Beaverton, OR, USA). The Python scripts are provided in the Supplementary Document.

## 4. Conclusions

In conclusion, sex disparity in opioid-associated adverse events was investigated by analyzing reports in FAERS. We identified 5,709,646 adverse events associated with 29 of the 30 studied opioids. Overall, more adverse events have been reported for women than men. Sex disparities for pairs between the 29 opioid drugs and 27 SOCs of adverse events were examined, and their statistical significances were evaluated. An analysis of all adverse events for the opioids demonstrated that three opioids—codeine, meperidine, and propoxyphene—not only have large numbers of adverse events reported in FAERS but also exhibit high sex disparities biased to women. Close examinations of sex disparities and statistical significance across the 29 opioids and 27 SOCs revealed that codeine, fentanyl, tapentadol, hydrocodone, and sufentanil showed significant sex disparities in many types of adverse events.

## Figures and Tables

**Table 1:** Adverse event reports identified from FAERS for the 30 opioid drugs.

Drug Number	Drug	Year to the Market	Adverse Event Reports	
			Women	Men
1	Alfentanil	1979	803	536
2	Alvimopan	2008	269	298
3	Buprenorphine	1981	103,684	97,300
4	Butorphanol	1979	828	218
5	Codeine	1832	190,152	42,405
6	Dihydrocodeine	1943	3868	3672
7	Fentanyl	1968	175,971	122,068
8	Hydrocodone	1920	191,885	200,209
9	Hydromorphone	1926	203,854	185,442
10	Levorphanol	1953	464	219
11	Loperamide	1976	32,600	16,995
12	Meperidine	1939	30,421	7990
13	Methadone	1947	48,637	63,519
14	Methylnaltrexone bromide	2008	1745	1085
15	Morphine	1817	308,480	249,095
16	Nalbuphine	1979	559	430
17	Naldemedine	2017	701	637
18	Nalmefene	1995	213	222
19	Naloxegol	2014	5096	3123
20	Naloxone	1971	69,655	62,880
21	Naltrexone	1984	34,345	38,042
22	Oxycodone	1917	954,050	768,829
23	Oxymorphone	1959	17,567	17,674
24	Pentazocine	1964	1176	499
25	Propoxyphene	1957	17,799	8032
26	Remifentanil	1996	4106	4235
27	Sufentanil	1984	5742	4476
28	Tapentadol	2008	12,780	7537
29	Tramadol	1972	214,331	143,481
30	Benzhydrocodone	2018	0	0

**Table 2:** Adverse event reports identified from FAERS for the 27 System Organ Classes (SOCs).

SOC Number	SOC Name	Adverse Event Reports	
		Women	Men
1	Blood and lymphatic system disorders	12,269	9016
2	Cardiac disorders	81,296	61,478
3	Congenital, familial, and genetic disorders	4072	5975
4	Ear and labyrinth disorders	8630	4747
5	Endocrine disorders	11,530	6011
6	Eye disorders	26,123	14,464
7	Gastrointestinal disorders	198,553	98,600
8	General disorders and administration site conditions	533,907	419,259
9	Hepatobiliary disorders	16,235	14,983
10	Immune system disorders	65,793	12,564
11	Infections and infestations	33,421	11,240
12	Injury, poisoning, and procedural complications	234,813	249,431
13	Investigations	102,697	52,561
14	Metabolism and nutrition disorders	14,226	10,200
15	Musculoskeletal and connective tissue disorders	153,561	55,111
16	Neoplasms benign, malignant, and unspecified	9050	7039
17	Nervous system disorders	109,830	82,187
18	Pregnancy, puerperium, and perinatal conditions	64,928	24,490
19	Product issues	19,143	11,967
20	Psychiatric disorders	568,680	691,940
21	Renal and urinary disorders	26,764	18,633
22	Reproductive system and breast disorders	10,037	9273
23	Respiratory, thoracic and mediastinal disorders	150,344	79,153
24	Skin and subcutaneous tissue disorders	94,238	22,621
25	Social circumstances	31,187	37,403
26	Surgical and medical procedures	24,341	23,895
27	Vascular disorders	26,113	16,907

## Data Availability

The original contributions presented in this study are included in the article/Supplementary Materials. Further inquiries can be directed to the corresponding author.

## Supplementary Materials

The following supporting information can be downloaded at: https://www.mdpi.com/article/10.3390/ph19040526/s1, Table S1: 30 opioid active ingredients and synonyms, Table S2: Opioid-related adverse event reports from the FDA Adverse Event Reporting System. Columns include active ingredient name (Active_Ingredient_in_Dictionary). Table S3: log_2_ of the ratio of adverse events reported for women to those reported for men. For cases no adverse events were reported for women and the ratio was zero, we assigned a log_2_ values of −5, which is lower than the minimum value of −4.6 observed in our data. Similarly, if there were no adverse events reported for men, the ratio was considered infinite, and we assigned a log_2_ value of 6, which is higher than the maximum value of 5.1 observed. If neither gender had any adverse event reported, we assigned a log_2_ value of zero.
